# Vaccination Enhances Early Immune Responses in White Shrimp *Litopenaeus vannamei* after Secondary Exposure to *Vibrio alginolyticus*

**DOI:** 10.1371/journal.pone.0069722

**Published:** 2013-07-22

**Authors:** Yong-Chin Lin, Jiann-Chu Chen, Wan Zabidii W. Morni, Dedi Fazriansyah Putra, Chien-Lun Huang, Chang-Che Li, Jen-Fang Hsieh

**Affiliations:** Department of Aquaculture, College of Life Sciences, National Taiwan Ocean University, Keelung, Taiwan, Republic of China; University of Cape Town, South Africa

## Abstract

**Background:**

Recent work suggested that the presence of specific memory or some form of adaptive immunity occurs in insects and shrimp. Hypervariable pattern recognition molecules, known as Down syndrome cell adhesion molecules, are able to mount specific recognition, and immune priming in invertebrates. In the present study, we attempted to understand the immune response pattern of white shrimp *Litopenaeus vannamei* which received primary (PE) and secondary exposure (SE) to *Vibrio alginolyticus*.

**Methodology:**

Immune parameters and proliferation of haematopoietic tissues (HPTs) of shrimp which had received PE and SE to *V. alginolyticus* were measured. In the PE trial, the immune parameters and proliferation of HPTs of shrimp that received heat-killed *V. alginolyticus* (HVa) and formalin-inactivated *V. alginolyticus* (FVa) were measured. Mortality, immune parameters and proliferation of HPTs of 7-day-HVa-PE shrimp (shrimp that received primary exposure to HVa after 7 days) and 7-day-FVa-PE shrimp (shrimp that received primary exposure to FVa after 7 days) following SE to live *V. alginolyticus* (LVa) were measured. Phagocytic activity and clearance efficiency were examined for the 7∼35-day-HVa-PE and FVa-PE shrimp.

**Results:**

HVa-receiving shrimp showed an earlier increase in the immune response on day 1, whereas FVa-receiving shrimp showed a late increase in the immune response on day 5. The 7-day-FVa-PE shrimp showed enhancement of immunity when encountering SE to LVa, whereas 7-day-HVa-PE shrimp showed a minor enhancement in immunity. 7-day-FVa-PE shrimp showed higher proliferation and an HPT mitotic index. Both phagocytic activity and clearance maintained higher for both HVa-PE and FVa-PE shrimp after 28 days.

**Conclusions:**

HVa- and FVa-receiving shrimp showed the bacteria agglutinated prior to being phagocytised. FVa functions as a vaccine, whereas HVa functions as an inducer and can be used as an immune adjuvant. A combined mixture of FVa and HVa can serve as a “vaccine component” to modulate the immunity of shrimp.

## Introduction

Vibriosis, which usually occurs when host animals become weakened, is considered a secondary and opportunistic infection, and incidences of vibriosis are associated with increases in *Vibrio* populations in culture pond waters [1], [2]. Therefore, prevention of disease outbreaks is a major concern in shrimp farming. Antibiotics and other therapeutic agents are widely used as common therapies in post-infection practice. However, application of these therapeutic chemicals has caused the development and spread of antibiotic resistance, reduced the efficacy of antibiotic treatments, caused residue accumulations in tissues and potential environmental hazards, and also raised public health issues [3], [4].

Immunoprophylaxis such as immunostimulation, vaccinations with materials of plant and microbial origin, and probiotics have become promising and feasible ways to prevent pathogenic infections [5]–[8]. Immunostimulants are known to induce innate immunity, increase the resistance against pathogens, and buffer immune depression, whereas vaccinations provide protection against specific pathogens. Vaccines can be prepared with formalin, ethanol, sodium sulphate, ammonium sulphite, fatty acids, and heating to inactivate the pathogen [9]. Several commercial vaccines have been developed for teleosts and shrimp based on the adaptive immunity or the presence of specific immune memory [10], [11].

Shrimp, like other invertebrates, rely solely on the innate immune system in which circulating haemocytes (hyaline cells (HCs), semi-granular cells (semi-GCs), and GCs) play a crucial role in defending against hostile microorganisms for self-protection [12]–[14]. It is known that the haematopoietic tissue (HPT) is responsible for producing and supplying haemocytes [15]. The immune system of shrimp consists of a pattern-recognition system, a prophenoloxidase (proPO)-activating system, the release of antimicrobial peptides and lysozymes, as well as phagocytosis, nodule formation, and encapsulation [16], [17]. proPO is activated to phenoloxidase (PO) by an endogenous trypsin-like serine proteinase through a serine proteinase cascade leading to melanin formation [18], [19]. Respiratory bursts (RBs) occur during phagocytosis and lead to the release of superoxide anions and other reactive oxygen species (ROS) [20]. Superoxide anions are scavenged by superoxide dismutase (SOD) to prevent damage to the host itself through an antioxidant system [21].

A vaccination with formalin-inactivated *Vibrio* sp. was effective against vibriosis in larval culture of penaeid shrimp [7], [8], [22]–[25]. Shrimp that received formalin-inactivated *V. harveyi* or a commercial vaccine that contained inactivated *Vibrio* exhibited enhanced phagocytosis [11], [26]. Administration of formalin-inactivated white spot syndrome virus (WSSV) or the purified envelope protein of WSSV produced a protective response against the virus in shrimp [27]–[30]. Recent work suggested that the presence of specific memory within the innate immune system or some form of adaptive immunity occurs in insects and shrimp [17], [31]–[34]. Furthermore, a Down syndrome cell adhesion molecule, also known as Dscam (a member of the immunoglobulin super family) which plays an important role in “alternative adaptive immunity” was observed in crustacean [35]–[37]. However, little or nothing is known about the mechanism of action of shrimp that receive formalin-inactivated pathogens, and the immune response of shrimp that encounter secondary infection with specific pathogens.

We assumed that shrimp which received formalin-inactivated *V. alginolyticus* (FVa) may show a different response to that of shrimp which received heat-killed *V. alginolyticus* (HVa), and assumed that shrimp which received FVa may show a secondary immune response pattern, similar to that which occurs in the adaptive immune response, and may show a different immune response after receiving a secondary infection of live *V. alginolyticus* (LVa). Accordingly, the present study examined immune parameters of shrimp that received LVa, HVa, and FVa, and examined survival rates and immune parameters of shrimp that received HVa and FVa after 7 days, and then received LVa and a post-LVa challenge. Haemocyte proliferation and the mitotic index of HPTs of control shrimp, HVa-receiving shrimp, and FVa-receiving shrimp were examined before and after LVa challenge. Haemograms, PO activities, RBs, SOD activities, and lysozyme activities were used as indicators of immune parameters [38]. Phagocytosis and clearance of shrimp that had received HVa and FVa after 7∼35 days were received a challenge with LVa. In addition, phagocytosis and clearance of shrimp that had received HVa and FVa after 7∼28 days were received a challenge with live *Bacillus subtilis* (LBs).

## Materials and Methods

### White Shrimp *Litopenaeus vannamei*

White shrimp *L. vannamei,* obtained from the University Marine Animal Center adjacent to the coast at Keelung, Taiwan, were shipped to our laboratory. Shrimp were placed in indoor fibreglass circular tanks containing aerated seawater (with a salinity of 35%), and acclimated in the laboratory for 3 weeks before experimentation began. During the acclimation period, shrimp were fed twice daily with a formulated shrimp diet (Tairou Feed Company, Tainan, Taiwan) at 3% of their body weight.

### Preparation of LVa and LBs

A pathogenic strain of *V. alginolyticus* isolated from diseased *L. vannamei* was used for the study [39]. The bacterium was cultured in tryptic soy broth (TSB supplemented with 2% NaCl, Difco, Sparks, MD, USA) for 24 h at 28°C, and then centrifuged at 7155×*g* for 20 min at 4°C [40]. The supernatant was removed, and the bacterial pellet was re-suspended in marine saline (MS) at 1.9×10^7^ and 4.0×10^8^ colony-forming units (cfu) ml^−1^ as respective bacterial suspensions to examine immune parameters and for the challenge test. *Bacillus subtilis* (BCRC10448) obtained from Bioresource Collection and Resource Center (Hsinchu, Taiwan) was cultured similarly as described above, and the bacterial suspension was prepared at 4.0×10^8 ^cfu ml^−1^ for the challenge test.

### Preparation of HVa and FVa

The bacterial suspension (1.9×10^7^ cfu ml^−1^) was centrifuged at 4500×*g* for 10 min at 4°C, and the bacterial pellet was washed with MS and centrifuged again to remove the culture medium. Finally, the pellet was resuspended in the same volume of MS and autoclaved at 121°C for 20 min. The autoclaved bacterial suspension was used for the heat-killed treatment. Another bacterial suspension (1.9×10^7^ cfu ml^−1^) was centrifuged, and the pellet was obtained similarly to that described above. The bacterial suspension in MS was supplemented with formalin to a final concentration at 1% formalin and incubated at 4°C for 24 h. The bacterial suspension was centrifuged at 4500×*g* for 10 min at 4°C to recover the bacterial pellet, then washed with MS and centrifuged again to remove the residual formaldehyde. The formalin-treated bacterial suspension was used for formalin-inactivated treatment.

### Experimental Design

**Figure 1:**
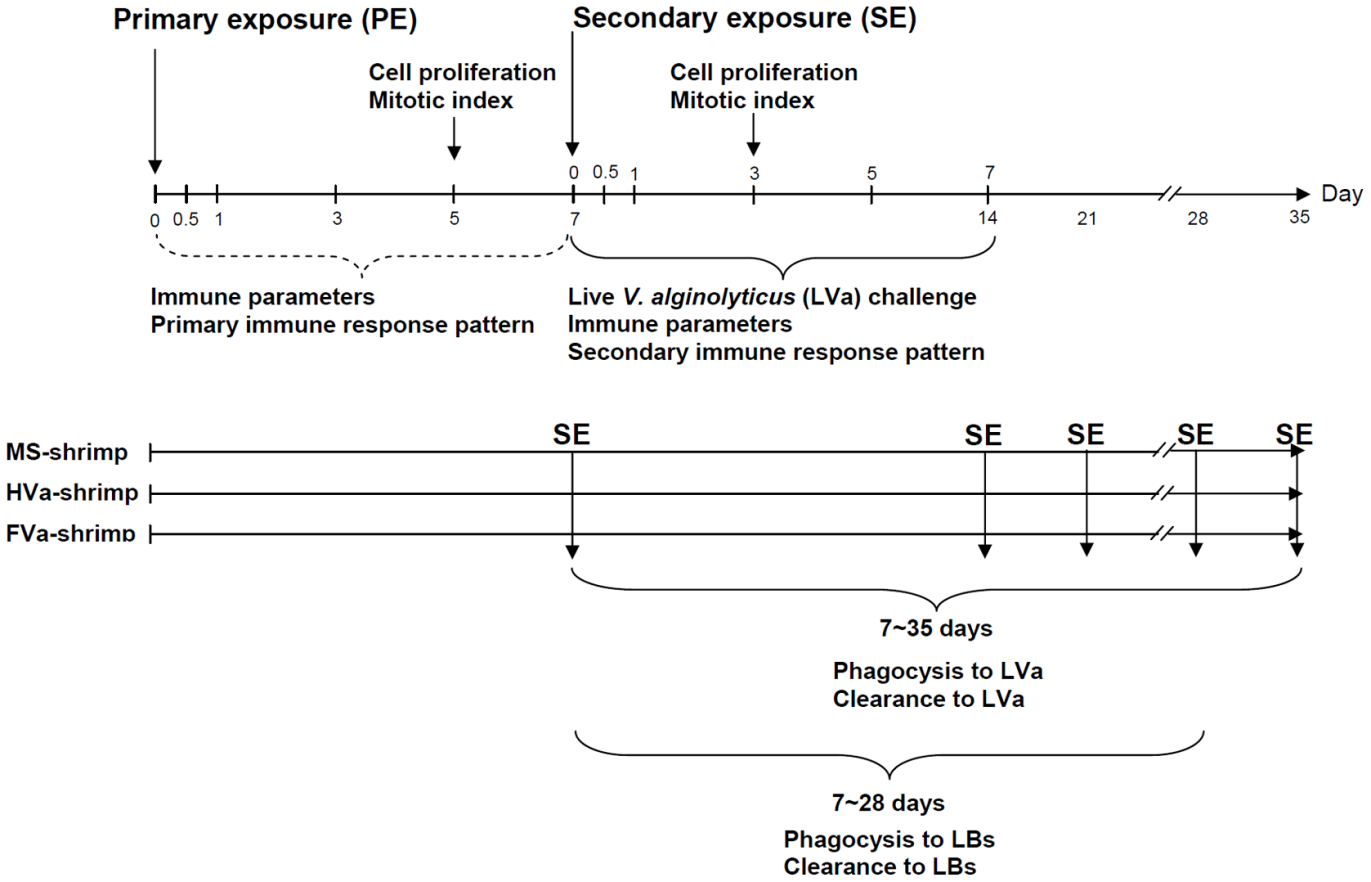
Experimental design diagram showing 1) primary exposure of white shrimp *Litopenaeus vannamei* that received heat-killed *Vibrio alginolyticus* (HVa) and shrimp that received formalin-inactivated *V.* *alginolyticus* (FVa), 2) showing secondary exposure of live *V. alginolyticus* (LVa) in shrimp that had received HVa and FVa after 7 days, and 3) showing phagocytosis and clearance to LVa and live *Bacillus subtilis* (LBs) in both 7∼35-day-HVa-PE and 7∼35-day-FVa-PE shrimp, and 7∼28-day-HVa-PE shrimp and 7∼28-day-PE shrimp.

Seven studies were conducted. Experiments were designed based on classical immunization to test primary exposure (PE) and secondary exposure (SE) to a specific pathogen (Fig. 1). (1) Immune parameters of control shrimp, MS-PE shrimp, LVa-PE shrimp, HVa-PE shrimp, and FVa-PE shrimp were examined for 0∼7 days. (2) Haemocyte proliferation and the mitotic index of HPTs in control, HVa-PE, and FVa-PE shrimp were determined on day 5. (3) Resistance of control, 7-day-HVa-PE shrimp (shrimp that received primary exposure to HVa after 7 days), and 7-day-FVa-PE shrimp (shrimp that received primary exposure to FVa after 7 days) were examined after SE to LVa. (4) Immune parameters of control, 7-day-HVa-PE, and 7-day-FVa-PE shrimp at 0.5∼7 days were determined after SE to LVa. (5) Haemocyte proliferation and the mitotic index of HPTs in control, 7-day-HVa-PE, and 7-day-FVa-PE shrimp were examined 3 days after SE to LVa. (6) Agglutination, phagocytosis and clearance in 7∼35-day-HVa-PE shrimp and 7∼35-day-FVa-PE shrimp that received a SE to LVa. (7) phagocytosis and clearance in 7∼28-day-HVa-PE shrimp and 7∼28-day-FVa-PE shrimp that receive a challenge with LBs. The weight of shrimp ranged 10.21∼12.63 g, and averaged 11.42±1.21 g (mean ± SD). There was no significant size difference among treatments. Only shrimp in the intermoult stage were used for the study. The moulting stage was determined by examining the uropoda, in which partial retraction of the epidermis could be distinguished [41]. During the experimental period, water temperatures ranged 26∼29°C, pH 7.89∼8.27, salinities 33‰∼35‰, and dissolved oxygen (DO) 5.68∼6.84 mg L^−1^.

### Immune Parameters of LVa-PE, HVa-PE, and FVa-PE Shrimp

There were five treatments, and each treatment was conducted at five different times (at 0, 0.5, 1, 3, 5, 7 days). There were (1) control shrimp, and (2) shrimp that received MS, (3) LVa, (4) HVa, and (5) FVa. Eight shrimp were used or each treatment and each time. Into the ventral sinus of the cephalothorax of each shrimp, 20 µl of a bacterial suspension (LVa, HVa, or FVa) at a concentration of 1.9×10^7^ cfu ml^−1^ was injected resulting in 3.8×10^5 ^cfu shrimp^−1^. Shrimp with no treatment served as the background control, and shrimp that received 20 µl of MS served as the positive control group. Haemolymph was withdrawn after 0.5, 1, 3, 5, and 7 days. Haemolymph sampling and preparation of diluted haemolymph followed previously described procedures [40]. Briefly, haemolymph (300 µl) was individually drawn from the ventral sinus of each shrimp, and diluted with 2700 µl of an anticoagulant solution. The haemolymph-anticoagulant mixture (diluted haemolymph) was placed in four tubes. The tubes contained 500, 1000, 1000, and 500 µl of diluted haemolymph, and were respectively used to measure (1) haemocyte counts and RBs, (2) PO activity, (3) SOD activity, and (4) lysozyme activity.

### Cell Proliferation and the Mitotic Index of HPT in Control, HVa-PE, and FVa-PE Shrimp at 5 days after PE

There were three treatments with five shrimp in each treatment. They were shrimp that had received (1) MS (control), (2) HVa, and (3) FVa. Shrimp were injected with MS, HVa, or LVa similarly to that described above. After 5 days, shrimp were sampled, and individually injected with vinblastine (V1377, Sigma) to inhibit mitosis. Briefly, shrimp were injected with 60 µl of vinblastine (0.5 mg ml^−1^) into the ventral sinus and released into normal seawater [15]. After 12 h, shrimp were injected with 3 ml of Davidson’s fixative solution (30 ml 95% ethanol, 20 ml 37% formaldehyde, 10 ml acetic acid, and 30 ml distilled water) into the dorsal part of the cephalothorax. Sampling of HPTs, fixation, embedding, preparation of permanent slides, and observation of HPTs followed previously described methods [15], [42], [43].

Other sets of HPT-containing slides were deparaffinised in xylene and rehydrated in a series of ethanol solutions and running tap water. A drop of RNase A solution (1 mg L^−1^) was placed on a slide for RNA digestion, followed by staining with a propidium iodide (PI) solution (0.2 mg ml^−1^) for 10 min, and then the slides were rinsed with tap water for about 45 min. Preparation of permanent slides, observations, and determination of the mitotic index of HPTs followed previously described methods [14], [42], [44].

### Resistance of Control, 7-day-HVa-PE, and 7-day-FVa-PE Shrimp Following SE to LVa

There were four treatments: one unchallenged treatment and three challenge treatments. Shrimp that received PE to (1) MS, (2) HVa after 7 days, and (3) FVa after 7 days, followed by SE to LVa served as the challenged groups. Into the ventral sinus of the cephalothorax of each shrimp, 20 µl of a bacterial suspension of HVa (1.9×10^7^ cfu ml^−1^) or FVa (1.9×10^7^ cfu ml^−1^) was injected resulting in 3.8×10^5^ cfu shrimp^−1^. For the challenged control group, shrimp were injected with an equal volume of a sterile MS. After 7 days, shrimp were individually injected into the ventral sinus of the cephalothorax with 20 µl of LVa at 4.0×10^8^ cfu ml^−1^ resulting in 8.0×10^6 ^cfu shrimp^−1^. Unchallenged control shrimp were injected with an equal volume of MS for the PE and with an equal volume of MS for the SE. There were 30 shrimp in each group. Experimental and control shrimp (10 shrimp aquarium^−1^) were kept in 40-L aquaria containing 20 L of seawater (35‰) with three replicates. Therefore, 120 shrimp [(3×3×10)+(1×3×10)] were used for the study. Survival of shrimp was examined every 12 h during the first day and then once a day after that until the end of experiment on day 7.

### Immune Parameter of Control, 7-day-HVa-PE, and 7-day-FVa-PE Shrimp Following SE to LVa

There were three treatments each of which was examined at five different time periods (0.5, 1, 3, 5, and 7 days). They were (1) shrimp that received MS (control shrimp), (2) shrimp that received HVa after 7 days (7-day-HVa-PE shrimp), and (3) shrimp that received FVa after 7 days (7-day-FVa-PE shrimp). Eight shrimp were used for each treatment and time. In addition, eight shrimp with no treatment were used for background values. Experimental shrimp which had been individually injected with 20 µl of HVa or FVa (1.9×10^7^ cfu ml^−1^) resulting in 3.8×10^5 ^cfu shrimp^−1^ were released into normal seawater. Control shrimp were injected with 20 µl of MS. After 7 days, shrimp were individually injected with 20 µl of LVa at 1.9×10^7^ cfu ml^−1^ resulting in 3.8×10^5^ cfu shrimp^−1^. Haemolymph was individually withdrawn after 0.5, 1, 3, 5 and 7 days after LVa challenge. Haemolymph sampling and preparation of diluted haemolymph were similar to those described above.

### Measurement of Immune Parameters

A drop of diluted haemolymph from the first tube was placed in a haemocytometer to measure HCs, GCs (including semi-GCs), and the total haemocyte count (THC) using an inverted phase-contrast microscope (Leica DMIL, Leica Microsystems, Wetzlar, Germany). The remainder of the diluted haemolymph mixture was used for subsequent tests.

The total PO activity was measured spectrophotometrically by recording the formation of dopachrome produced from L-dihydroxyphenylalanine (L-DOPA) as previously described [45] with some modifications. Briefly, from the second tube, 1000 µl of diluted haemolymph was centrifuged at 800×*g* and 4°C for 20 min. Details of the measurements were described previously [40].

RBs of haemocytes were quantified using the reduction of nitroblue tetrazolium (NBT) to formazan as a measure of superoxide anions, as previously described [43]. Briefly, 100 µl of diluted haemolymph from the first tube was placed in triplicate in a 96-well microplate previously coated with 100 µl of a poly-L-lysine solution (0.2%) to improve cell adhesion. Details of the measurements were described previously [40].

SOD activity was measured by its ability to inhibit superoxide radical-dependent reactions using a Ransod kit (Randox, Crumlin, UK). Briefly, 1000 µl of diluted haemolymph from the third tube was centrifuged at 800×*g* and 4°C for 20 min. Details of the measurement were described previously [40], [46].

Lysozyme activity was determined following a previously described method [47]. Briefly, 500 µl of diluted haemolymph from the fourth tube was centrifuged, and the precipitate was mixed with 1 ml (0.02%) of *Micrococcus lysodeikticus* (Sigma, St Louis, MO, USA). Details of the measurements were described previously [47], [48].

### Cell Proliferation and the Mitotic Index of HPTs of Control, 7-day-HVa-PE, and 7-day-FVa-PE Shrimp Following SE to LVa at 3 days after SE

There were three treatments with five shrimp in each treatment. They were shrimp that received PE to (1) MS, (2) HVa, and (3) FVa for 7 days. Eight shrimp were used for each treatment and time. Shrimp which had been individually injected with 20 µl of HVa or FVa (1.9×10^7^ cfu ml^−1^) resulting in 3.8×10^5 ^cfu shrimp^−1^ were released into normal seawater (35% salinity) for 7 days. Shrimp were then subjected to SE to LVa, and after 3 days, shrimp were sampled, and individually injected with vinblastine (V1377, Sigma) to inhibit mitosis. The following steps concerning the injection of vinblastine, HPT fixation, slide staining, preparation of permanent slides, and observations of cell proliferation and the mitotic index were conducted similarly to those described in Section 2.6.

### Phagocytosis and Clearance Efficiency in HVa-PE Shrimp and FVa-PE Shrimp that Received a SE to LVa, and Phagocytois and Clearance in HVa-PE Shrimp and FVa-PE Shrimp that Received a Challenge with LBs

For the SE to LVa, there were three treatments. Shrimp that had received PE to (1) MS, (2) HVa, and (3) FVa for 7, 14, 21, 28 and 35 days, and then received SE to LVa (3.8×10^5^ cfu shrimp^−1^) for 3 h were used for examination of agglutinated “bacteria cluster”, phagocytosis and clearance observations. Fifty microliter of haemolymph was sampled, gently mixed with 50 µl anticoagulant solution, and then followed by adding 100 µl fixative solution (1% paraformaldehyde) for 30 mins of incubation. The fixative haemocytes were spread on the slide glass using the cytospin machine (Thermo, Shandon, UK) at 1000 rpm, for 10 min. The slide glass were strained with Liu’s A solution for 30 sec, Liu’s B solution for 60 sec, and then washed with running water for 30 sec. Cells of *V. alginolyticus* of different treatments were stained similarly to those mentioned above. Examinations of phagocytic activity and clearance efficiency were conducted following the previously described methods [48]. Phagocytic activity, defined as the phagocytic rate (PR) was expressed as PR (%) = [(phagocytic haemocytes)/(total haemocytes)]×100. Phagocytic index (PI) was expressed as PI = [(bacteria phagocytized by phagocytiic hamocytes)/(phagocytic haemocytes)]. The number of bacterial colonies in the control shrimp (MS) expressed as the control group, and the colonies of shrimp that received HVa as well as shrimp that received FVa served as the test groups. The clearance efficiency was expressed as “100- [(cfu in test group)/(cfu in control group)] ×100”. For the comparison of LBs, there were three treatments. Shrimp that had received (1) MS, (2) HVa, and (3) FVa after 7, 14, 21, and 28 days, then received a challenge with LBs (3.8×10^5^ cfu shrimp^−1^) for 3 h were used for examination of phagocytosis and clearance observations. Phagocytic activity, phagocytic index, and clearance efficiency were conducted similarly as those described before.

### Statistical Analysis

All data were subjected to a one-way analysis of variance (ANOVA). If significant differences were indicated at the 0.05 level, then a multiple-comparisons (Tukey’s) test was used to examine significant differences among treatments using SAS computer software (SAS Institute, Cary, NC, USA). The percent data (resistance test) were normalized using arcsine-transformation before the analysis. Statistical significance of differences required that *p* be <0.05.

## Results

### Immune Parameters of LVa-PE, HVa-PE, and FVa-PE Shrimp

**Figure 2:**
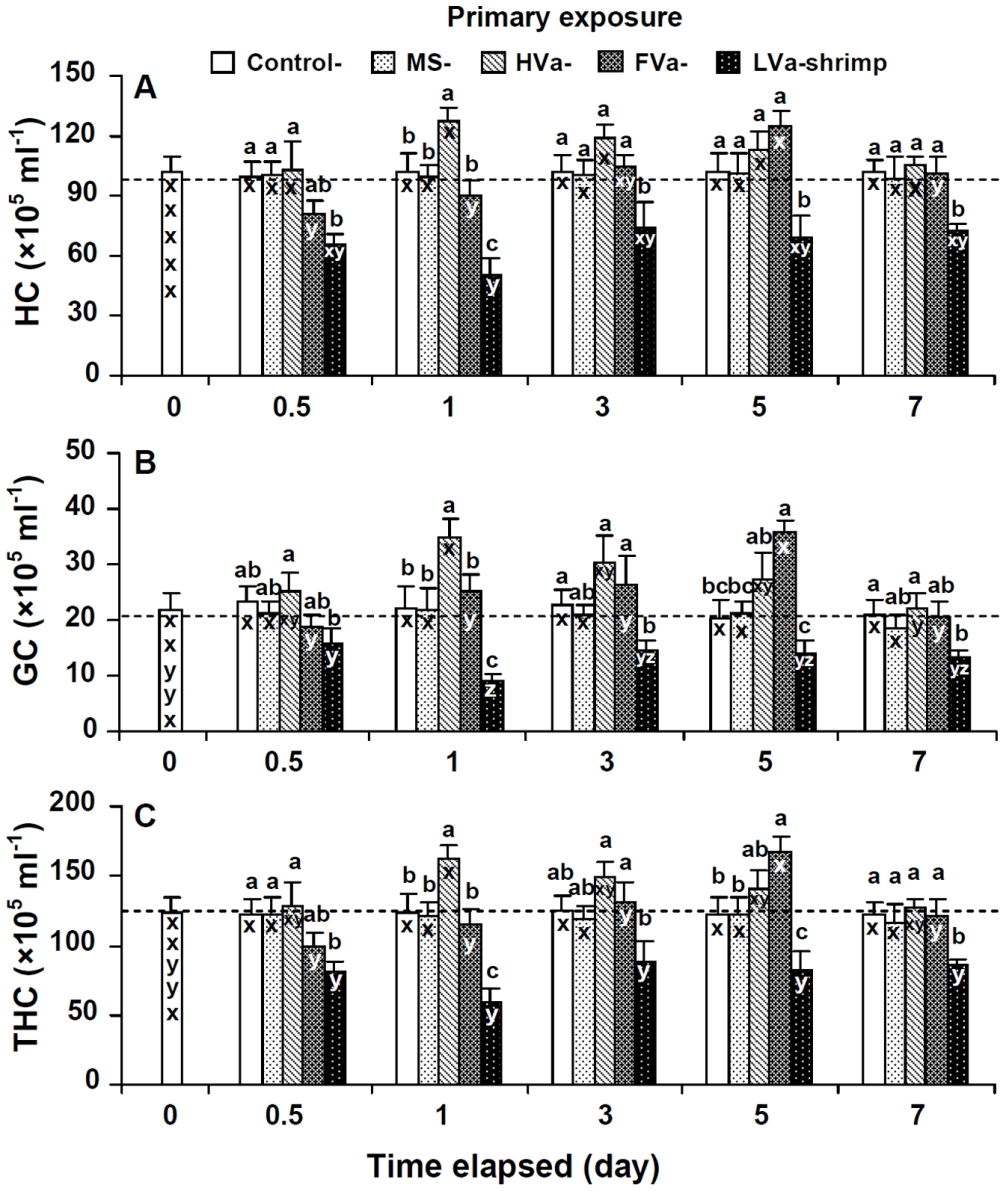
Hyaline cells (HCs) (A), granular cells (GCs) (including semi-GCs) (B), and the total haemocyte count (THC) (C) of control white shrimp *Litopenaeus vannamei*, and shrimp that received marine saline (MS), heat-killed *Vibrio alginolyticus* (HVa), formalin-inactivated *V.* *alginolyticus* (FVa), and live *V. alginolyticus* (LVa) after 0, 0.5, 1, 3, 5 and 7 days. Each bar represents the mean from six shrimp with the standard error (SE). Data at the same exposure time with different letters (a, b, c) significantly differ (*p*<0.05) among treatments. Data at the same treatment with different letter (x, y, z) significantly differ (*p*<0.05) among different elapsed time periods (days).

No significant differences in HCs, GCs, THCs, PO activities, RBs, SOD activities, or lysozyme activities were observed in control shrimp and shrimp that received MS among the different time periods. HCs, GCs, and THCs of shrimp that received LVa decreased after 0.5 days, reached the lowest level after 1 day, and then remained lower than background values for 0.5∼7 days. HCs, GCs, and THCs of shrimp that received HVa increased for 1∼5 days, and then returned to background values after 7 days. HCs, GCs, and THCs of shrimp that received FVa decreased at day 0.5, but then increased afterwards, and increased to the highest level at day 5 (Fig. 2).

**Figure 3:**
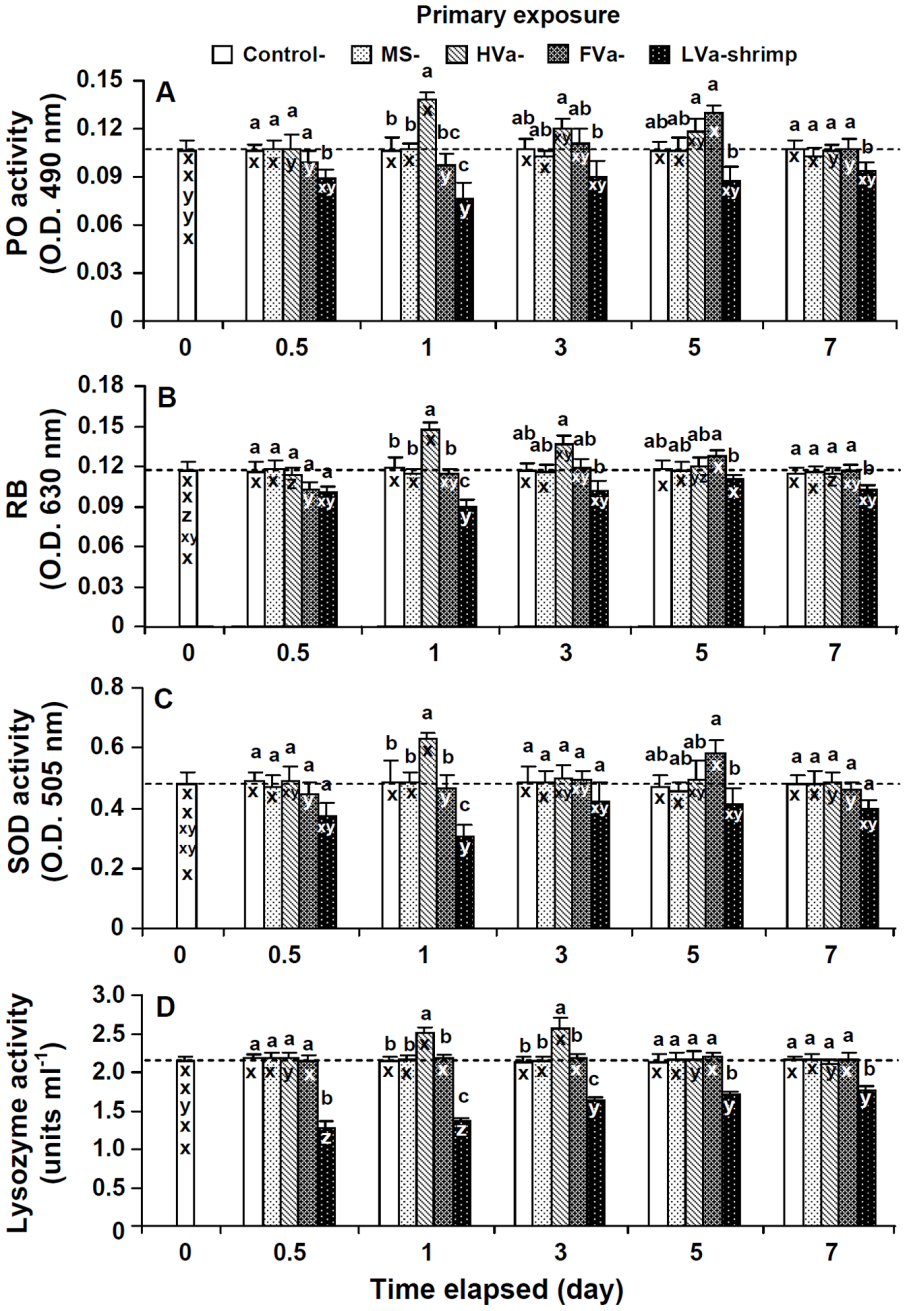
Phenoloxidase (PO) activity (A), respiratory bursts (RBs) (B), superoxide dismutase (SOD) activity (C) and lysozyme activity (D) of control white shrimp *Litopenaeus vannamei*, and shrimp that received marine saline (MS), heat-killed *Vibrio alginolyticus* (HVa), formalin-inactivated *V.* *alginolyticus* (FVa), and live *V. alginolyticus* (LVa) after 0, 0.5, 1, 3, 5 and 7 days. See Fig. 2 for statistical information.

PO activities, RBs, SOD activities, and lysozyme activities of shrimp that received LVa decreased, and were lower than background values at 0.5∼7 days. PO activities, RBs, SOD activities, and lysozyme activities of shrimp that received HVa significantly increased after 1 day, then slightly decreased afterwards, and remained similar to background values after 7 days. PO activities, RBs, and SOD activities of shrimp that received FVa decreased at day 0.5, increased afterwards to the highest level at day 5, but had returned to background values by day 7. Lysozyme activities of shrimp that received FVa remained unchanged during 0.5∼7 days (Fig. 3).

**Figure 4:**
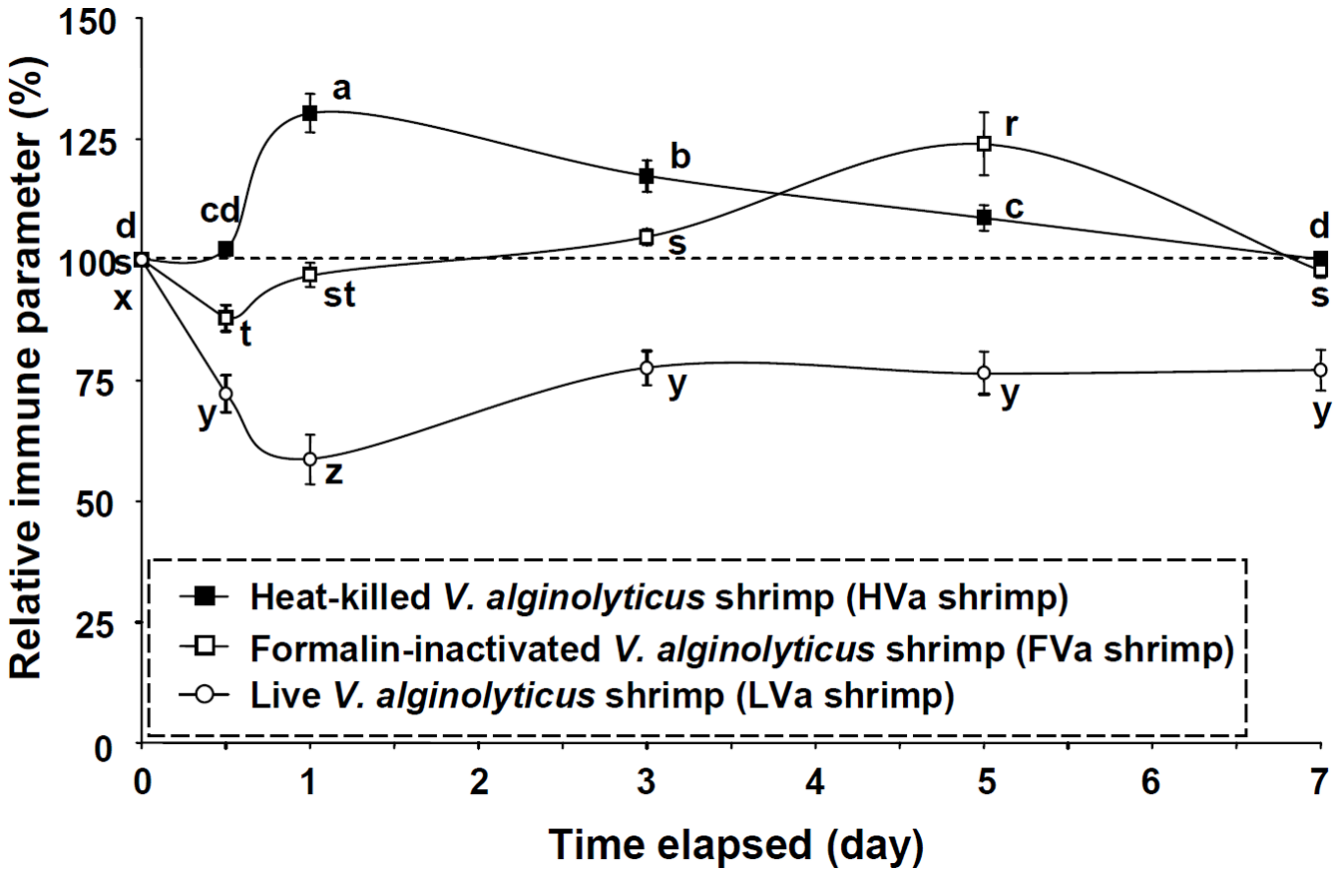
Relative immune parameters (%) of white shrimp *Litopenaeus vannamei* that received heat-killed *Vibrio alginolyticus* (HVa), formalin-inactivated *V.* *alginolyticus* (FVa), and live *V. alginolyticus* (LVa) after 0.5, 1, 3, 5 and 7 days. Data (mean ± SE) with different letters (a, b, c) in shrimp that received HVa, with different letters (r, s, t) in shrimp that received FVa, and with diffferent letters (x, y, x) in shrimp that received LVa significantly differ (*p*<0.05) among different elapsed time periods (days).

In order to reveal innate immune response patterns of shrimp which received PE to HVa, FVa, and LVa, immune parameters were normalized to their background values, and are presented as relative percentage values (Fig. 4). Immune parameter patterns of shrimp that received LVa decreased during 0.5∼7 days. Immune parameter patterns of shrimp that received HVa increased sooner at day 1, then gradually decreased, and had returned to background values by day 7. Immune parameter patterns of shrimp that received FVa decreased at day 0.5, then increased afterwards, and reached the highest values at day 5. Shrimp which received HVa, FVa and LVa exhibited different innate immune response patterns.

### Cell Proliferation and the Mitotic Index of HPTs in HVa-PE and FVa-PE Shrimp at day 5 after PE

**Figure 5:**
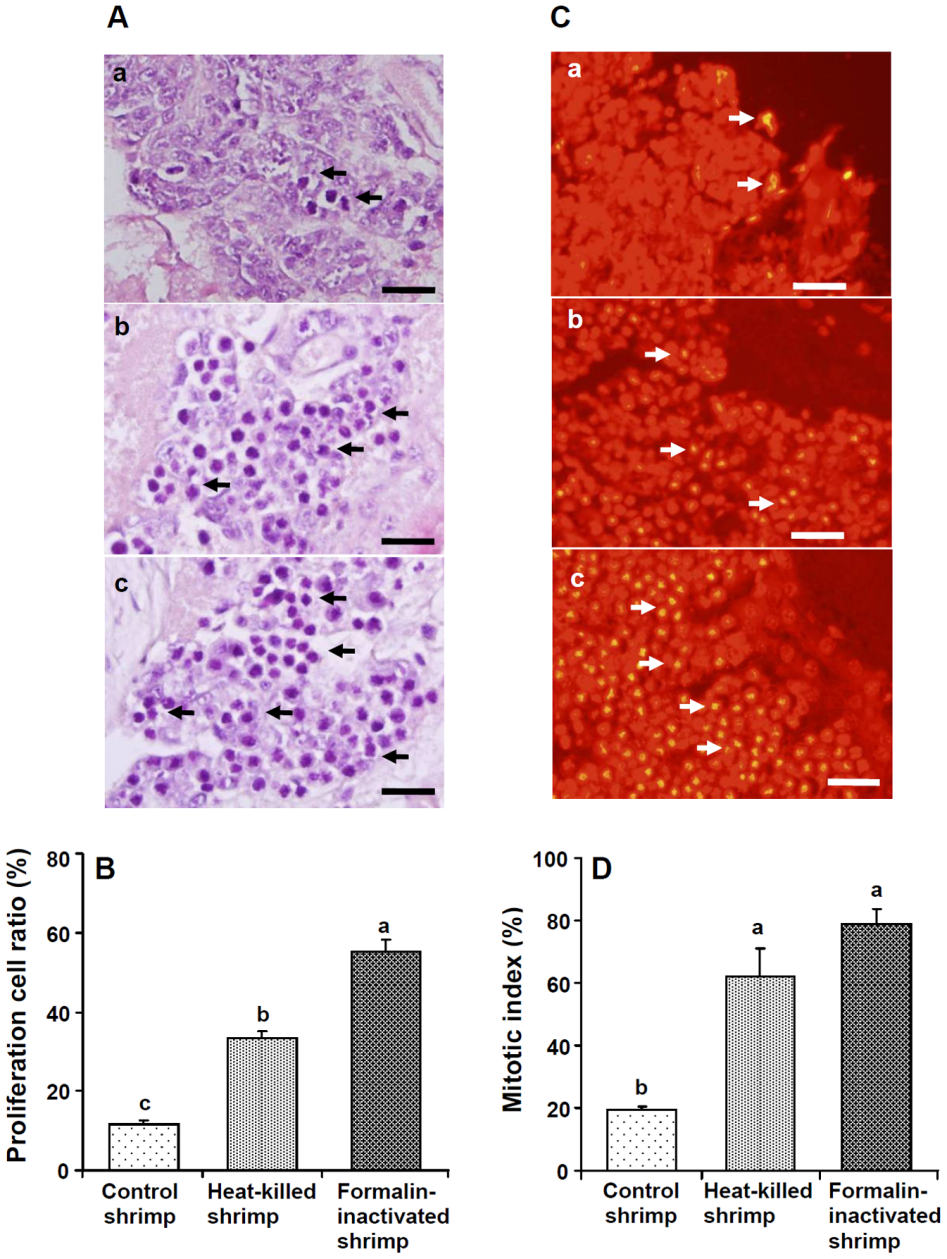
(A) Optical micrographs of H&E-stained haematopoietic tissues (HPTs) of control shrimp (a), and shrimp that received heat-killed *Vibrio alginolyticus* (HVa) (b) and formalin-inactivated *V.* *alginolyticus* (FVa) (c) after 5 days. Higher numbers of mitotic cells (arrows) were observed in shrimp that received HVa and shrimp that receive FVa. (B) Proliferation cell ratios (%) in HPTs of shrimp that received FVa and shrimp that received HVa were significantly higher than that of control shrimp. (C) Fluorescent micrographs of propidium iodide-stained HPTs of control shrimp (a), shrimp that received HVa (b), and shrimp that received FVa (c) after 5 days. Higher numbers of mitotic cells (arrows) were observed in shrimp that received HVa and shrimp that received FVa. (D) The mitotic index, expressed as a percentage of dividing cells in HPTs of shrimp that received FVa and those that received HVa was significantly higher than that of control shrimp. Each bar represents the mean value from six different images with the standard error. Data with different letters significantly differ (*p*<0.05) among treatments. Scale = 20 µm.

Optical photographs of H&E-stained HPTs of control shrimp, shrimp that received HVa, and shrimp that received FVa after 5 days are shown in Fig. 5A. Proliferation cell ratios of shrimp that received HVa and those that received FVa significantly increased (Fig. 5B). Fluorescent photographs of PI-stained HPTs of control shrimp, shrimp that received HVa, and shrimp that received FVa after 5 days are shown in Fig. 5C. Higher numbers of mitotic cells (arrows) were observed for shrimp that received HVa and those that received FVa. The mitotic index, expressed as the percentage of dividing cells in HPTs, was significantly higher in shrimp that received HVa and those that received FVa than that of control shrimp (Fig. 5D).

### Resistance of Control, 7-day-HVa-PE, and 7-day-FVa-PE Shrimp Following SE to LVa

**Figure 6:**
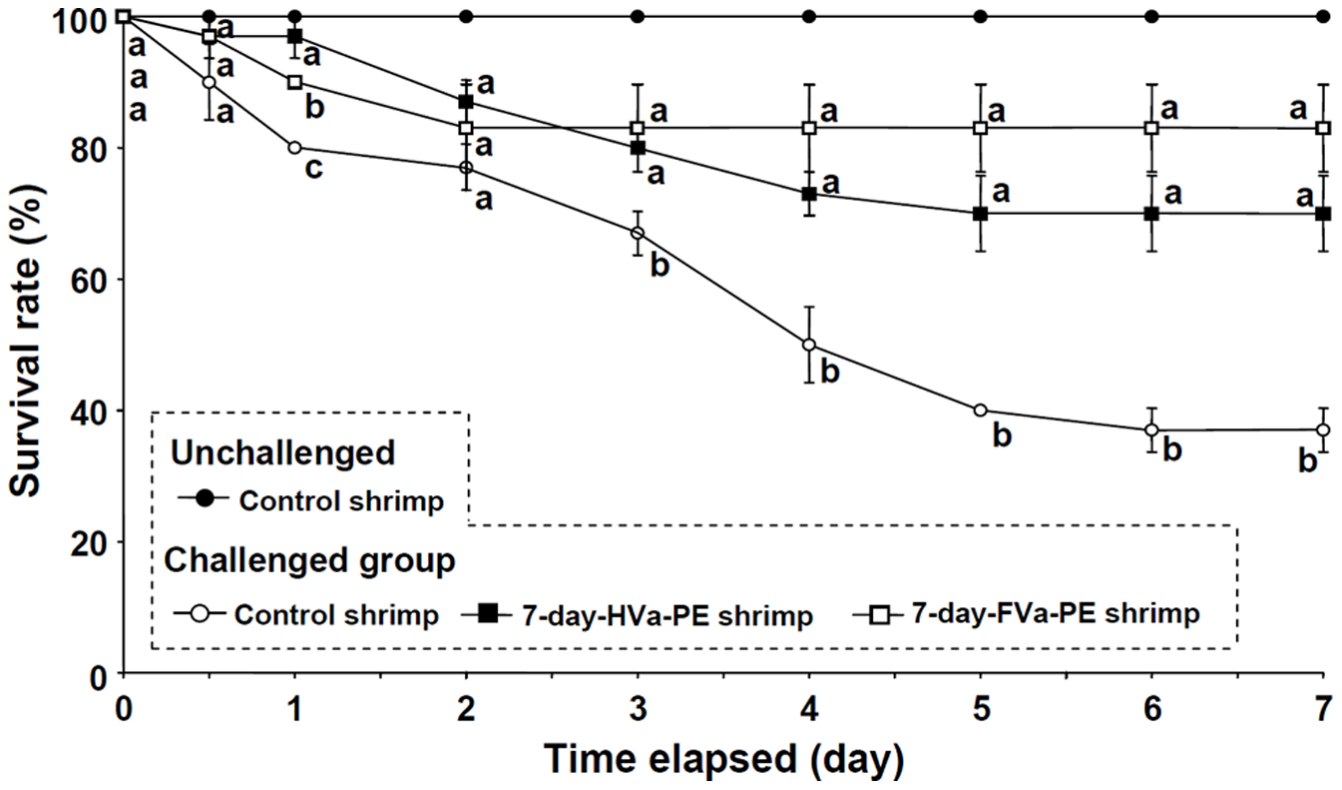
Survival rates of unchallenged control white shrimp *Litopenaeus vannamei*, control shrimp, and shrimp that received heat-killed *Vibrio alginolyticus* (HVa) and formalin-inactivated *V.* *alginolyticus* (FVa) after 7 days, and then challenged with live *V. alginolyticus* (LVa) at a dose of 8.0×10^6^ cfu shrimp^−1^. Each data point represents the mean of three replicates (10 shrimp in each replicate) with the standard error (SE). Data with different letters in the same time period significantly differ (*p*<0.05) among treatments.

All unchallenged control shrimp survived. In contrast, death began to occur after 0.5 days in the challenged groups. No significant difference in survival rates at 3∼7 days was observed between shrimp that received HVa and those that received FVa. However, the survival rate was higher (83.3%) in shrimp that received FVa followed by shrimp that received HVa (70%) at 5∼7 days. The survival rate of control shrimp was significantly lower, and was 40% at 4∼7 days after challenge (Fig. 6).

### Immune Parameters of Control, 7-day-HVa-PE, and 7-day-FVa-PE Shrimp Following SE to LVa

**Figure 7:**
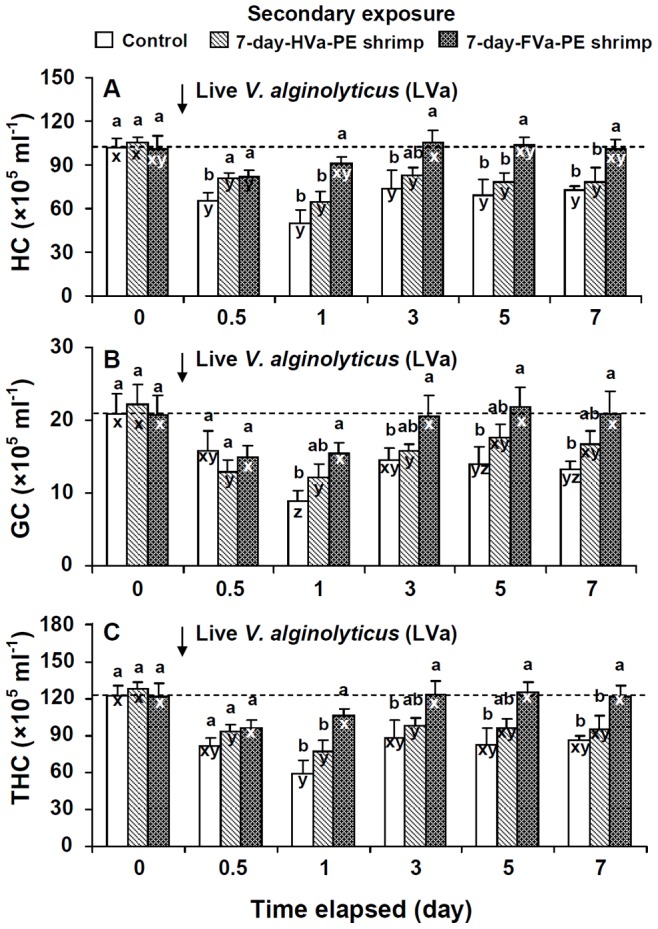
Hyaline cell (HC) (A), granular cells (GCs) (including semi-GCs) (B), and total haemocyte count (THC) (C) of control white shrimp *Litopenaeus vannamei*, and shrimp that received heat-killed *Vibrio alginolyticus* (HVa) and formalin-inactivated *V.* *alginolyticus* (FVa) after 7 days, and then were challenged with *V. alginolyticus* at a dose of 3.8×10^5^ cfu shrimp^−1^ after 0, 0.5, 1, 3, 5 and 7 days. See Fig. 2 for statistical information.

**Figure 8:**
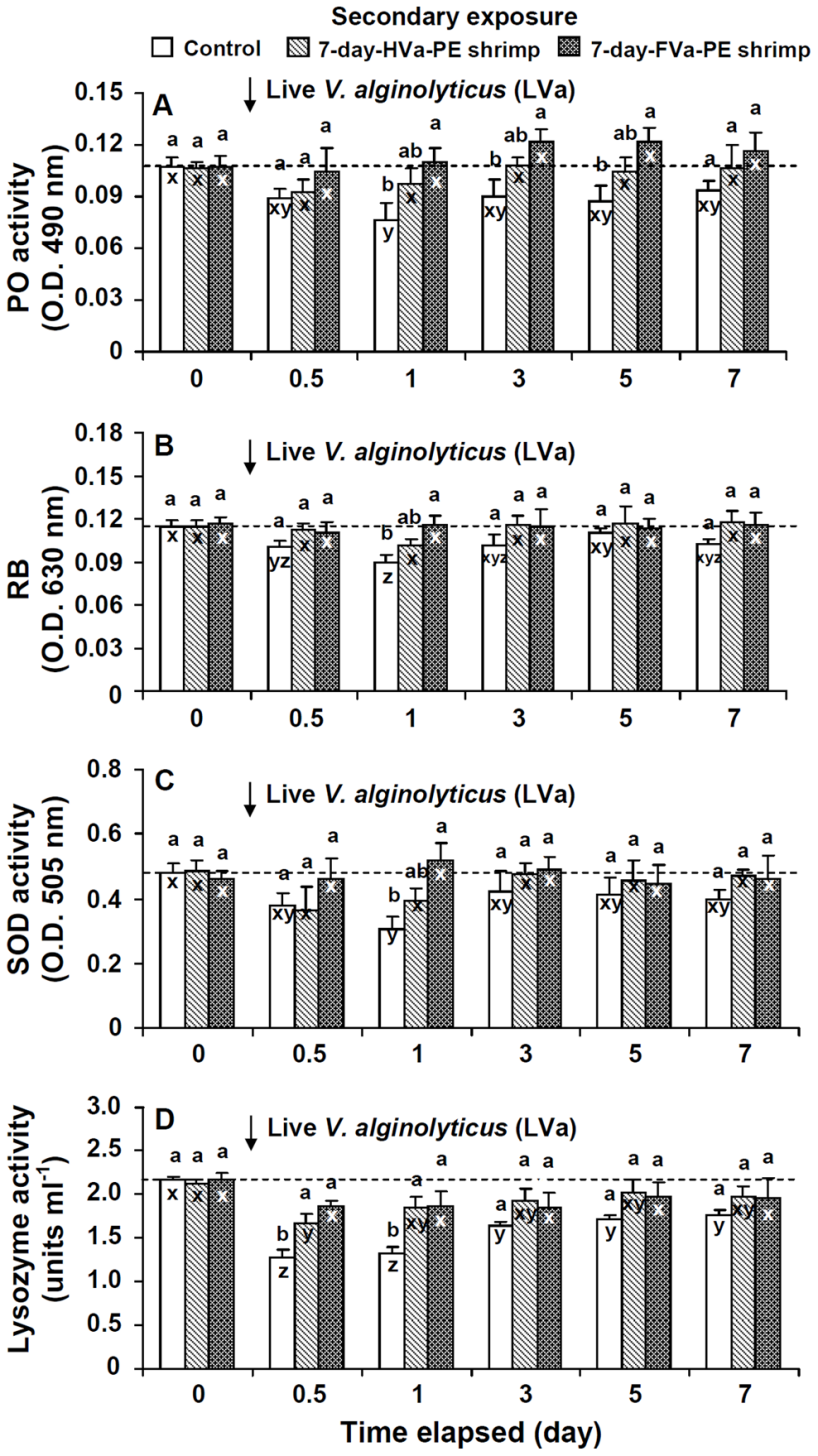
Phenoloxidase (PO) activity (A), respiratory bursts (RBs) (B), superoxide dismutase (SOD) activity (C), and lysozyme activity (D) of control white shrimp *Litopenaeus vannamei*, and shrimp that received heat-killed *Vibrio alginolyticus* (HVa), and formalin-inactivated *V.* *alginolyticus* (FVa) after 7 days, and then were challenged with *V. alginolyticus* at a dose of 3.8×10^5^ cfu shrimp^−1^ after 0, 0.5, 1, 3, 5 and 7 days. See Fig. 2 for statistical information.

Immune parameters of control shrimp decreased at days 0.5∼7 after SE to LVa. Immune parameters of 7-day-HVa-PE shrimp gradually increased at days 3∼7 after SE to LVa, but values still remained lower than background values. HCs, GCs, and THCs of 7-day-FVa-PE shrimp were lower at days 0.5∼1 after SE to LVa, then increased thereafter, and remained similar to background values at days 3∼7 after SE to LVa. PO, RB, SOD, and lysozyme activities of 7-day-FVa-PE shrimp increased at day 1 after SE to LVa, and values remained similar to background values at days 0.5∼7 after SE to LVa (Figs. 7, 8).

**Figure 9:**
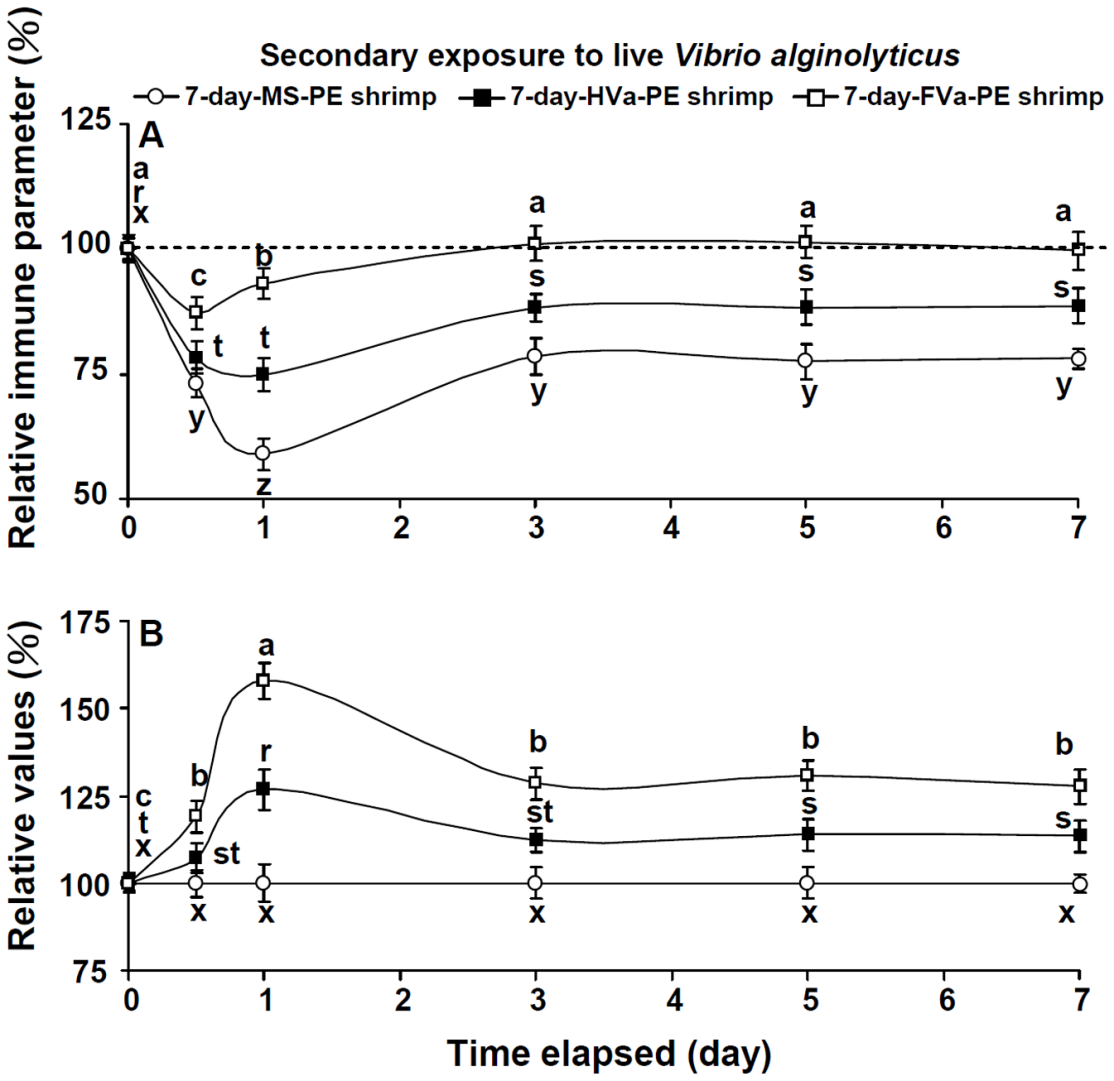
(A) Relative immune parameter (%) of control white shrimp *Litopenaeus vannamei*, and shrimp that received heated-killed *Vibrio alginolyticus* (HVa) and formalin-inactivated *V.* *alginolyticus* (FVa) after 7 days and then were challenged with live *V. alginolyticus*. Data (mean ± SE) with different letters (x, y, z) in control shrimp, with different letters (r, s, t) in shrimp that received HVa, and with different letters (a, b, c) in shrimp that received FVa significantly differed (*p*<0.05) among elapsed times (day). (B) Relative values of HVa-receiving shrimp and FVa-receiving shrimp to that of control shrimp. Data with different leters (r, s, t) in HVa-receiving shrimp, and with different letters (a, b, c) in FVa-receiving shrimp significantly differ (p<0.05) among elapsed times (day).

In order to reveal secondary immune response patterns of shrimp that received PE to MS, HVa, and FVa, followed by SE to LVa, immune parameters were normalized to their background values, and are presented as relative percent values (Fig. 9A). Immune parameter patterns of control and 7-day-HVa-PE shrimp decreased to the lowest at day 1 after SE to LVa, then gradually increased, but values remained much lower than background values at 1∼7 days after SE to LVa. However, immune parameter patterns of 7-day-FVa-PE shrimp decreased at day 0.5, gradually increased at day 1, and values remained similar to background values at days 3∼7 days after SE to LVa. A further calculation was performed to normalize relative values of 7-day-FVa-PE and 7-day-HVa-PE shrimp to control shrimp (Fig. 9B). The immunity of both 7-day-HVa and 7-day-FVa shrimp was provoked when they encountered SE to LVa, but 7-day-FVa shrimp showed a more-efficient immune response than did 7-day-HVa shrimp.

### Cell Proliferation and the Mitotic Index of HPTs in Control, 7-day-HVa-PE, and 7-day-FVa-PE Shrimp Following SE to LVa at day 3 after SE

**Figure 10:**
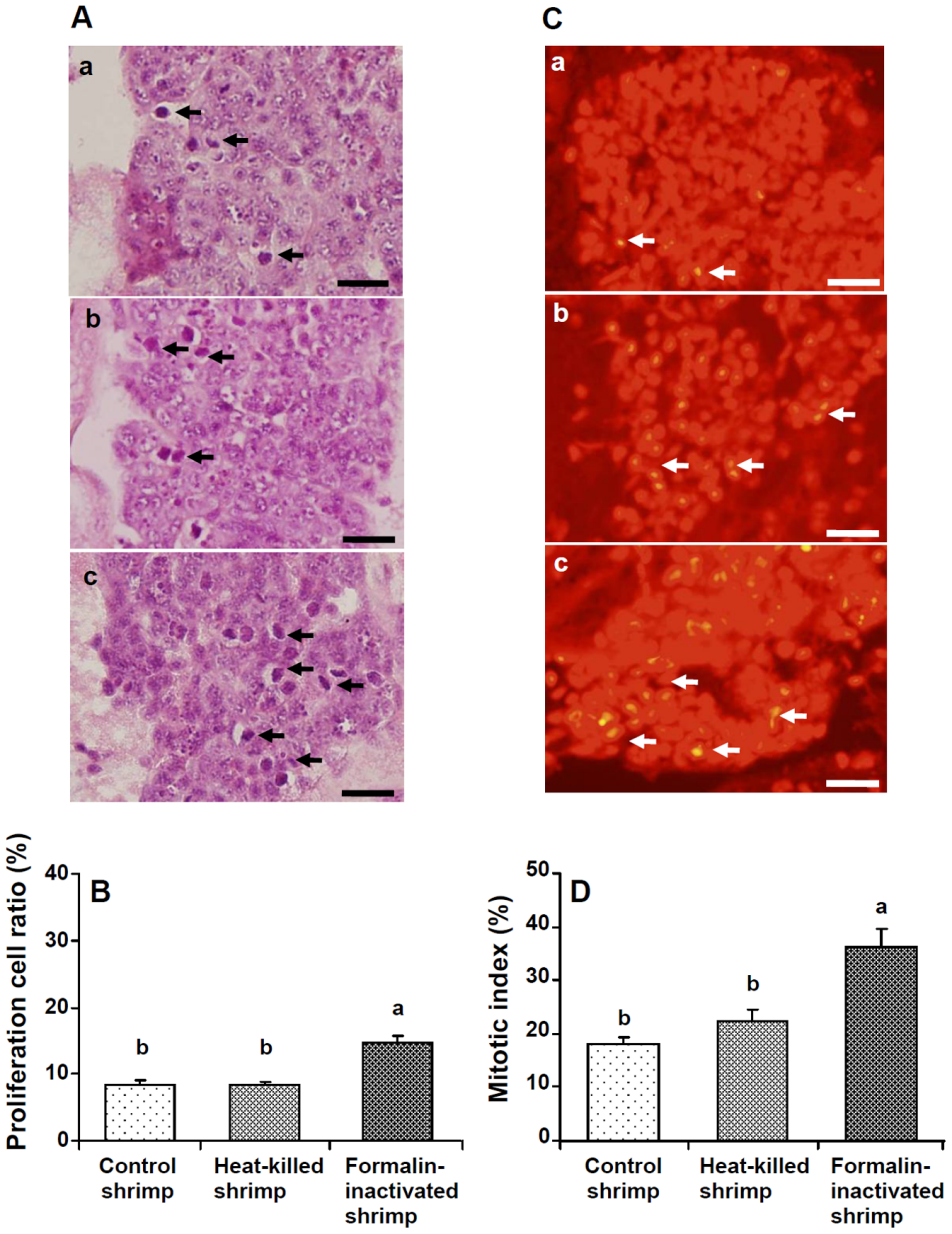
(A) Optical micrographs of H&E-stained haematopoietic tissues (HPTs) of control shrimp (a), shrimp that received heat-killed *Vibrio alginolyticus* (HVa) (b), and formalin-inactivated *V.* *alginolyticus* (FVa) (c) after 7 days and then received live *V. alginolyticus* (LVa) after 3 days. Higher numbers of mitotic cells (arrows) were observed in shrimp that received FVa. (B) The proliferation cell ratio (%) in HPTs of shrimp that received FVa after 7 days and then challenged with LVa after 3 days was significantly higher. (C) Fluorescent micrographs of propidium iodide-stained HPTs of control shrimp (a), and shrimp that received HVa (b) and FVa (c) after 7 days, and then were challenged with LVa after 3 days. Higher numbers of mitotic cells (arrows) were observed in shrimp that received FVa. (D) The mitotic index, expressed as a percentage of dividing cells in HPTs of shrimp that received FVA after 7 days, and then challenged with LVa after 3 days was significantly higher. Scale = 20 µm. See Fig. 5B for statistical information.

Optical photographs of H&E-stained HPTs of control, 7-day-HVa-PE, and 7-day-FVa-PE shrimp followed by SE to LVa at day 3 after SE are shown in Fig. 10A. The proliferation cell ratio of 7-day-FVa-PE shrimp was significantly higher than those of 7-day-HVa-PE and control shrimp following SE to LVa (Fig. 10B). Fluorescent photographs of PI-stained HPTs of control, 7-day-HVa-PE, and 7-day-FVa-PE shrimp following SE to LVa are shown in Fig. 10 C. Higher numbers of mitotic cells (arrows) were observed in shrimp that received FVa. The mitotic index was significantly higher in 7-day-FVa-PE shrimp than in control and 7-day-HV-PE shrimp at 3 days after SE (Fig. 10D).

### Phagocytosis and Clearance Efficiency in HVa-PE Shrimp and FVa-PE Shrimp that Received a SE to LV, and Phagocytois and Clearance in HVa-PE Shrimp and FVa-PE Shrimp that Received a Challenge with LBs

**Figure 11:**
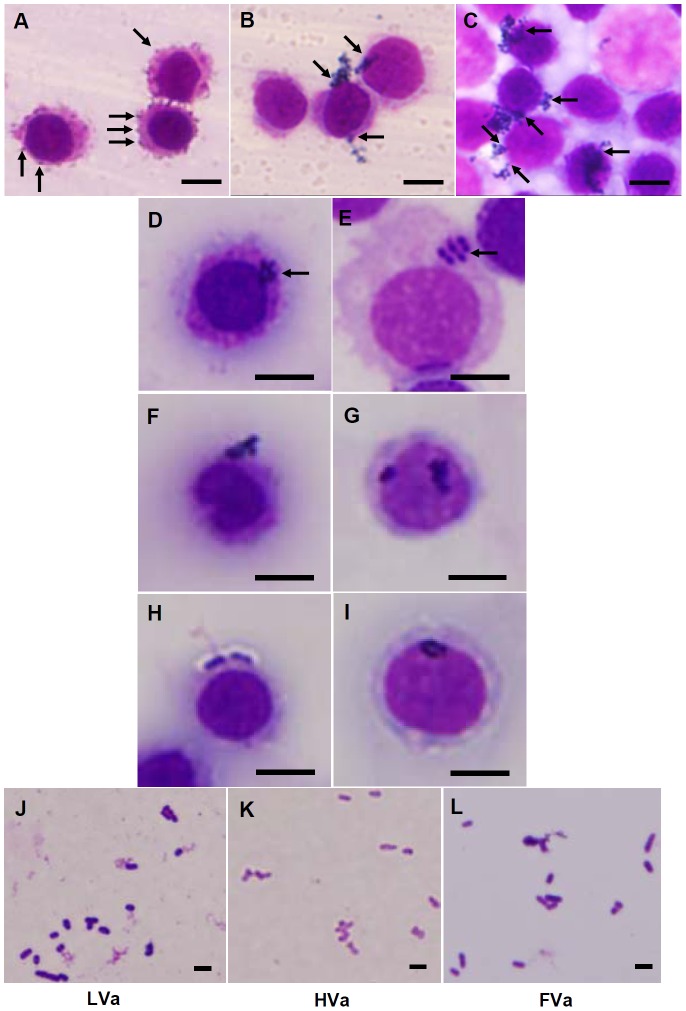
Agglutinative activity of control white shrimp (A), shrimp that received heat-killed *Vibrio alginolyticus* (HVa) (B), and shrimp that received formalin-inactivated *V.* *alginolyticus* (FVa) (C) after 7 days, and then were subjected to live *V. alginolyticus* (LVa). Shrimp that received FVa and HVa showed higher agglutinative activities. Agglutinated “bacteria cluster” phagocytized by hyaline cells (HC) (D) and semi-granular cell (SGC) (E) were observed in both 7-day-HVa-PE shrimp and 7-day-FVa-PE shrimp, by both HC (F) and SGC (G) in 14-day-HVa-PE shrimp and 14-day-PE shrimp, and by both HC (H) and SGC (I) in 21-day-Hva-PE shrimp and 21-day-FVa-PE shrimp, and then received SE. The intact of different *V. alginolyticus* including LVa (J), HVa (K), and FVa (L) were strained with Liu’s straining. Scale = 5 µm (A∼I), and 2 µm (J∼L).

The haemocyte staining of control shrimp, 7-day-HVa-PE shrimp, and 7-day-FVa-PE shrimp and then received LVa after 3 h was shown in Figs. 11A–11C. In the HVa and FVa treatments, bacteria agglutinated to form bacterial clusters, and then attached to the haemocytes. In both treatments, bacterial clusters phagocytosized by HC and SGC were observed (Figs. 11D, 11E). Similar phenomenon of bacterial agglutination and phagocytic HC and SGC was observed in both 14-day-HVa-PE shrimp and 14-day-FVa-PE shrimp (Figs. 11F, 11G), and both 21-day-HVa-PE and 21-day-FVa-PE shrimp (Figs. 11H, 11I). The intact of LVa, HVa, and FVa was confirmed with Liu’s staining (Figs. 11J–11L).

**Figure 12:**
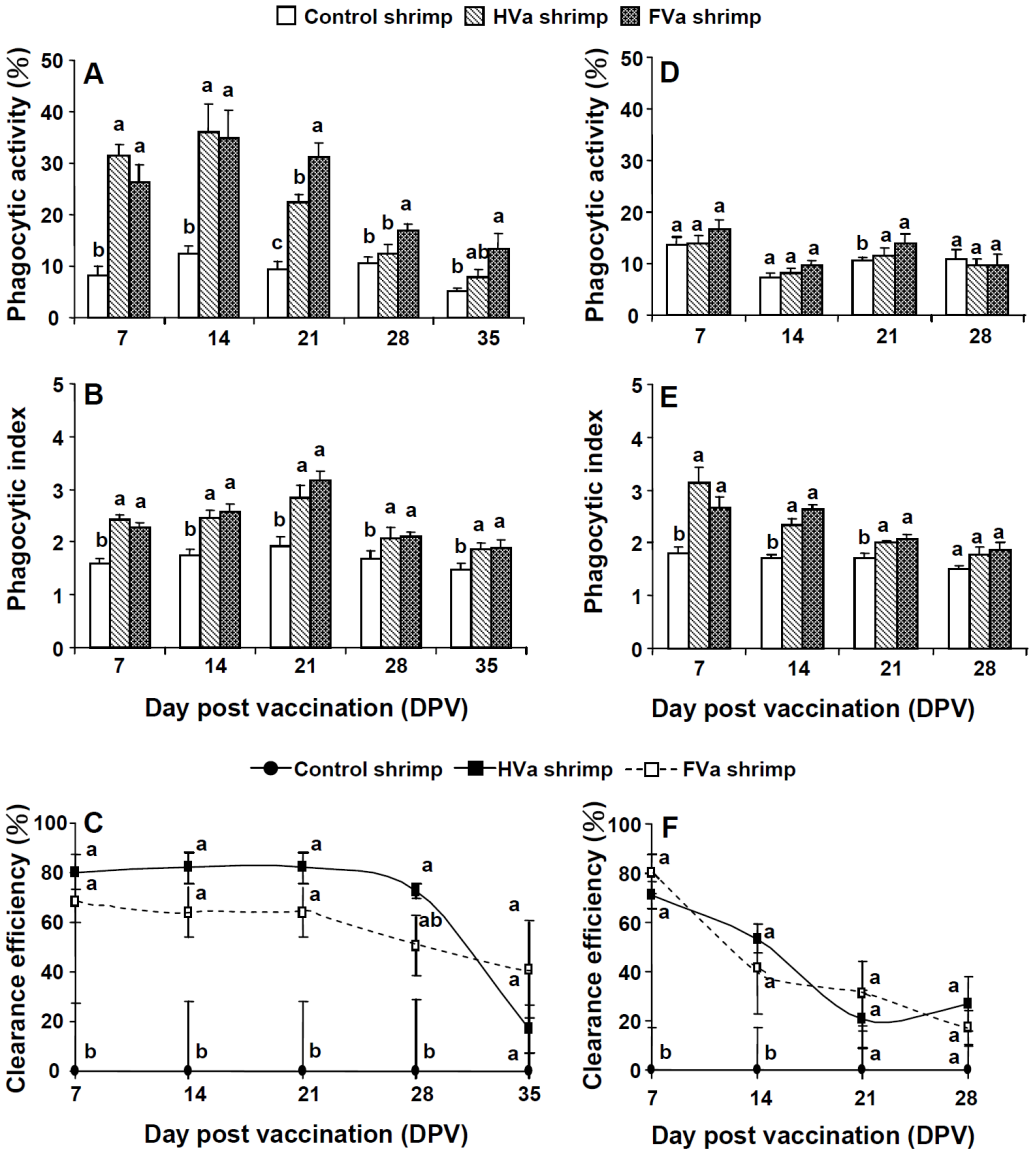
Phagocytic activity (A), phagocytic index (B), and clearance efficiency (C) of control shrimp, 7∼35-day-HVa-PE shrimp, and 7∼35-day-FVa-PE shrimp and then received live *Vibrio alginolyticus* (LVa). Phagocytic activity (D), phagocytic index (E), and clearance efficiency (F) of control shrimp, 7∼28-day-HVa-PE shrimp, and 7∼28-day-FVa-PE shrimp and then received live *Bacillus sublitis* (LBs). Data at the same exposure time with different letter (a, b, c) significantly differ (*p*<0.05) among different treatments.

Phagocytic activities of 7∼35-day-FVa-PE shrimp and 7∼21-day-HVa shrimp received SE to LVa were significantly than those of control shrimp that received LVa (Fig. 12A), whereas phagocytic indices of 7∼35-day-FVa-PE shrimp and 7∼35-HVa-PE shrimp received SE to LVa were slightly higher than that of control shrimp that received LVa (Fig. 12B). Clearance efficiency of both treatments remained better performance up to 28 days (Fig. 12C). No significant difference in phagocytic activity was observed among the 7∼28-day-HVa, 7∼28-day-FVa, and control shrimp that received LBs (Fig. 12 D), whereas phagocytic indices of 7∼28-day-FVa-PE shrimp and 7∼28-HVa-PE shrimp received LBs were slightly higher than that of control shrimp that received LBs (Fig. 12E). Clearance efficiencies of 7-day-HVa-PE shrimp and 7-day-FVa-shrimp that received LBs were significantly higher than those of control shrimp that received LBs. However, the tendency of clearance efficiency decreased after 14 days (Fig. 12F).

## Discussion

Challenge with *V. anguillarum* and *V. alginolyticus* decreased haemocyte counts in tiger shrimp *Penaeus monodon* and white shrimp *L. vannamei* [15], [49], and a low-salinity stress more strongly decreased the haemocyte count of white shrimp *L. vannamei* which had received *V. alginolyticus* [50]. Haemocytes are primary immune effectors that carry out cell-mediated immunity including phagocytosis, coagulation, melanisation, and opsonisation in arthropods [16]. In the present study, an injection of LVa caused decreases in haemocyte counts and immune parameters at 0.5∼7 days post-injection. Phagocytic and apoptotic haemocytes were observed in white shrimp *L. vannamei* challenged with *V. alginolyticus* [51], [52]. Haemocyte cunts dramatically change, and that reflect the shrimp’s immunity. The immune response, which is like wrestling between the host immunity and a pathogen, is a kinetic response that depends on the superior status of the host immunity or pathogen. Once the pathogen begins proliferating or escapes from the host defence, it can cause immune failure in the host. However, once the host immunity gets the upper hand, the host may recover from the pathogenic infection. Decreases and recoveries of haemocyte counts and other immune parameters vary greatly with the dose of *V. alginolyticus* and the health status of shrimp. Furthermore, previous research indicated that shrimp can recognize the specific bacteria in the secondary exposure [26]. In the present study, we conducted kinetic immune response of shrimp that received primary and secondary exposure to *V. alginolyticus*.

A pattern of an earlier decline of the haemocyte count within a few hour followed by a slight recovery or an increase in the haemocyte count was observed in several species of shrimp and crab challenged with microbe-related polysaccharides such as lipopolysaccharide (LPS) and β-glucan [53], [54]. Enhanced earlier recovery of haemocytes was observed in the white shrimp *L vannamei* which received *Gracilaria tenuistipitata* extract and then was subjected to a *V. alginolyticus* challenge [40]. White shrimp that received carrageenan, *G. tenuistipitata* extract, β-glucan, and fucoidan via an injection showed increases in the haemocyte count and other immune parameters after 1 day [55], [56]. In the present study, the haemocyte count and other immune parameters of shrimp that received HVa also increased after 1 day, but values of these parameters gradually returned to background levels after 7 days. The immune response pattern of shrimp that received HVa was earlier (after 1 day), and was similar to the pattern of shrimp that received other immunostimulants like carrageenan, β-glucan, fucoidan, and *G. tenuistipitata* extract. It is suggested that HVa functions as an inducer for earlier immunity, which is similar the functioning of those other immunostimulants.

In the present study, shrimp that received FVa showed much higher immune parameters and resistance than did shrimp which received HVa at 1∼7 days after SE to *V. alginolyticus* (Figs. 6, 9A, 9B), and showed much higher proliferation and a mitotic index of HPTs at 3 days after SE (Fig. 10). These facts indicate that the immune performance of FVa-receiving shrimp was more efficient than that of HVa-receiving shrimp when encountering a SE to *V. alginolyticus*. Both heat and formalin treatments of bacteria can be used to develop vaccine preparations, but the efficiency of the vaccine may vary depending on the amount of antigenicity retained [9]. Heat conditions for vaccine preparation might be not consistent for every species of bacteria. Overheating may disrupt the antigen conformation, reduce its antigenicity, and disrupt bacterial cell membranes leading to the release of membrane-bound polysaccharides. HVa caused an earlier immune response (Fig. 4) and a minor enhancement of immune response with SE (Fig. 9B). Furthermore, less staining difference was observed among LVa, HVa and FVa in appearance (Figs. 11J–11L). The cell of HVa which stained in more pink-red indicated that the surface protein still interacted with cell wall, but the inner content may be lost. This fact indicates that HVa may retain less antigenicity but released more LPS, leading to its performance as an immunostimulant rather than a vaccine.

Activation of the innate immune system and responses to FVa, HVa, and LVa by shrimp showed different primary immune patterns (Fig. 4). Activation of the innate immune system and responses of 7-day-FVa-PE and 7-day-HVa-PE shrimp after SE to LVa also showed different secondary immune patterns (Figs. 9A, B). These facts suggest that the recognition and recall memory in HVa-receiving and FVa-receiving shrimp exhibited different recognition patterns and activated different responses. In the case of HVa preparation, the epitope of HVa is thought to be denatured, and disclosed more cell-bound polysaccharides which are more rapidly recognized by PRPs leading to an early immune reaction. However, FVa, which retains intact polysaccharide and protein components, is not more easily or efficiently recognized by PRPs than is HVa, leading to a late immune reaction. Specific recognition and immune education are thought to take more time (Fig. 4). FVa may have better antigenicity retention, and induce more hypervariable immunorecognition molecules against SE.

Recently, a Dscam which is encoded by hypervariable gene family showed that invertebrates are able to mount some form of specific immunity, termed “specific immune priming” [17], [57]. Elevated phagocytosis and proliferation of haemocytes could support a high degree of specificity and memory [17], [58]. Dscam, which has been found in shrimp, crayfish and insect, is known to mediate haemocyte and pathogen adhesion in vaccinated animals [35∼37, 59, 60]. In insect, the malaria *Anopheles gambiae* Dscam (AgDscam) is a hypervariable pattern recognition receptor (PRR) of innate immune system, and is mediated phagocytosis of bacteria with which it can associate and defend against in a splice form-specific memory [59]. In crustacean, the bacteria specific isoforms of crayfish *Pacifastacus leniusculus* Dscam (PiDscam) were shown to have a specific binding property to bacteria, and the bacteria specific isoforms of PiDscam were shown to be associated with bacterial clearance and phagocytosis in crayfish [37]. These studies carried out so far may have claimed some sort of specific immunity. However, further research is needed in providing mechanistic insight into possible mechanism and to confirm whether specific immunity does exist.

In the present study, shrimp that received PE to HVa and FVa showed higher cell proliferation and mitotic indices of HPTs, and also heightened phagocytic capabilities when they encountered SE to LVa. Furthermore, FVa and HVa enhanced the formation of *V. alginolyticus* clusters, which attached to HCs (Figs. 11B, 11C). The bacterial clusters phagocytosized by the haemocytes in the 7∼21-day-PE (Figs. 11D-11I), despite the image of engulfed *V. alginolyticus* was murky. By comparison, both HVa-PE shrimp and FVa-PE shrimp showed better performance in phagocytosis and clearance than control shrimp (Figs. 12A∼C). By integrative point of view in phagocytosis and clearance, better elimination ability of LVa occurred in the shrimp that had received HVa and FVa after 7∼28 days, and then received a LVa challenge, whereas slight elimination of LBs occurred in both shrimp that had received HVa and FVa after 7∼14 days, and then received a LBs challenge (Fig. 12). Similarly, slight elimination ability of live *Vibrio harveyi* (LVh) occurred in both shrimp that had received HVa and FVa after 7 days in response to LVh (data not shown). The recognition of shrimp that had received HVa and FVa showed different pattern when encountered LVa and LBs. Shrimp that had received HVa and FVa temporarily increased elimination performance in response to LBs is likely to show an immunostimulatory effect by the first receiving HVa and FVa. Furthermore, shrimp that had received HVa and FVa, and then received LVa may imply an existence of specific recognition and memory. Both FVa-receiving shrimp and HVa-receiving shrimp are able to restore the bacterial information into its immune system up to 28 days. It is suggested that the familiar *V. alginolyticus* may be agglutinated by specific recognition molecules like Dscam to form bacterial clusters which are easily and efficiently phagocytised [37], [59]. Both FVa and HVa can also induce immune responses by increasing the proPO and phagocytosis systems of shrimp. However, the functional mechanism is still unknown.

Despite that there is still a far away to develop a true shrimp vaccine; here we provided the data of immune response patterns of shrimp that encountered a primary and secondary exposure to *Vibrio alginolyticus*. FVa-receiving shrimp were considered to recognize cell surface characteristics of the same bacteria more efficiently than HVa-receiving shrimp, leading to earlier immune modulation with SE. Several scientists have claimed efficacious effects of resistance to *Vibrio* in shrimp following vaccination via immersion [11], [24], [26]. The phenomenon of “vaccination” was observed in the present study. However, the functional mechanism of specific recognition, haemocyte education in the HPTs and enhancement of immune parameters is needed for further study.

From the present study results, both HVa and FVa can be used as vaccines for protecting shrimp against SE to *V. alginolyticus.* FVa is a better candidate according to the primary immune pattern, the secondary immune pattern, heightened phagocytosis, and high strength in provoking immunity, whereas HVa showed two faces as both an immunostimulant and vaccine according to the earlier increase in the primary immune pattern, minor enhancement in the secondary immune pattern, heightened phagocytosis, and minor strength in provoking immunity. Therefore, HVa can be used as an “immune potentiator” or “adjuvant” that facilitates induction of specific recognition and memory. The combined mixture of FVa and HVa may work efficiently as a “vaccine component” to modulate immune responses and enhance the immune ability in the shrimp farming industry against vibriosis.

In conclusion, immune parameters of HVa-receiving shrimp increased earlier after 1 day, whereas immune parameters of FVa-receiving shrimp increased after 5 days, and both treatments showed higher HPT proliferation. FVa-receiving shrimp showed higher resistance against LVa, and showed earlier recovery of immune parameters and higher proliferation and mitotic index of HPTs at 3 days post-challenge. Shrimp may have specific memory based on the present study. The immune recognition of shrimp that received HVa and FVa may show a recall of memory up to 28 days. The application of a combined mixture of vaccine components of HVa (as an adjuvant) and FVa (as an antigen) is suggested to enhance the immunity of shrimp and its resistance against pathogenic infection in the shrimp farming industry.
